# Species-specific gamete recognition initiates fusion-driving trimer formation by conserved fusogen HAP2

**DOI:** 10.1038/s41467-021-24613-8

**Published:** 2021-07-19

**Authors:** Jun Zhang, Jennifer F. Pinello, Ignacio Fernández, Eduard Baquero, Juliette Fedry, Félix A. Rey, William J. Snell

**Affiliations:** 1 Department of Cell Biology and Molecular Genetics, University of Maryland, College Park, MD USA; 2 Unité de Virologie Structurale, Virology Department and CNRS UMR 3569, Institut Pasteur, Paris, France

**Keywords:** Membrane proteins, Cell adhesion, Fertilization

## Abstract

Recognition and fusion between gametes during fertilization is an ancient process. Protein HAP2, recognized as the primordial eukaryotic gamete fusogen, is a structural homolog of viral class II fusion proteins. The mechanisms that regulate HAP2 function, and whether virus-fusion-like conformational changes are involved, however, have not been investigated. We report here that fusion between *plus* and *minus* gametes of the green alga *Chlamydomonas* indeed requires an obligate conformational rearrangement of HAP2 on *minus* gametes from a labile, prefusion form into the stable homotrimers observed in structural studies. Activation of HAP2 to undergo its fusogenic conformational change occurs only upon species-specific adhesion between the two gamete membranes. Following a molecular mechanism akin to fusion of enveloped viruses, the membrane insertion capacity of the fusion loop is required to couple formation of trimers to gamete fusion. Thus, species-specific membrane attachment is the gateway to fusion-driving HAP2 rearrangement into stable trimers.

## Abstract (web summary)

HAP2 is essential for gamete fusion during fertilization and is conserved among eukaryotes. Here the authors show that species-specific adhesion between *Chlamydomonas plus* and *minus* gametes initiates HAP2 to undergo a fusogenic conformational change into homotrimers via a molecular mechanism akin to that of enveloped viruses.

## Introduction

Species-specific recognition between two gametes during fertilization is intimately linked in time and space with the gamete membrane fusion reaction. Remarkably, the biochemical reaction that drives gamete bilayer merger in even a single organism has not been established, and whether the temporal link between attachment and bilayer merger reflects mechanistic links is unknown. A number of candidate gamete recognition/adhesion proteins have been identified in protists, plants, and metazoans^1–7^, but none are broadly conserved. Conversely, the single-pass transmembrane protein HAP2 (also called GCS1) is essential for bilayer merger during gamete fusion in organisms from green algae to malaria-causing protozoan parasites, and from arthropods to flowering plants, and is likely the ancestral gamete fusogen^8–18^. The failure yet to identify proteins in chordates or fungi with homology to any HAP2 family member suggests either that HAP2 was replaced or that it has diverged sufficiently to make it unrecognizable by current methods.

X-ray structure studies^19–21^ and homology modeling^22,23^ show that HAP2 is a homolog of the class II fusion proteins of alphaviruses, flaviviruses, and bunyaviruses, which include the agents of important diseases in humans such as dengue, Zika, rubella, chikungunya and yellow fever. Class II viral proteins are structural elements of the viral envelopes and are essential for lipid bilayer fusion with the target cell during infection^24–26^. Depending on the virus type, the proteins are present as metastable hetero- or homo-dimers. Upon interaction with a target cell and endocytosis, the acidic environment of the endosome initiates structural changes in the fusion proteins that release them from the dimer, and expose a previously shielded, hydrophobic residue-rich “fusion loop” distal to the transmembrane domain. The fusion loops at the tip of the newly monomeric proteins insert into the endosomal membrane, thereby bridging the viral and target cell membranes. Continued structural re-organization, “hairpinning,” into the most energetically favored state, the folded-back trimer, drags the two membranes together and drives bilayer merger^24,25,27^

The structural studies using recombinant forms of the ectodomains of *Chlamydomonas* and *Arabidopsis* HAP2^19,20,28^ show the same trimer of hairpins conformation observed in the postfusion forms of the viral proteins. These studies, along with in vivo evidence indicating a role for the HAP2 fusion loop in fusion^19,21,29^, have led to the working model that HAP2 also transitions from a prefusion form on *minus* gametes into homo-trimers during the fusion reaction. A conflicting model based on studies of HAP2-dependent fusion in heterologous cells proposes formation of hetero-trimers composed of HAP2 on one gamete and HAP2 or another unidentified protein on the other gamete^23,30,31^. To date, however, there is no direct biochemical evidence that endogenous HAP2 in any organism even forms trimers, or that trimer formation by class II fusion proteins drives fusion in any eukaryote^32^.

Here, we report that a conformational rearrangement of HAP2 from a detergent-sensitive form in naive gametes into an SDS-resistant trimer indeed drives the gamete fusion reaction between mating type *minus* and mating type *plus* gametes of *Chlamydomonas*. Mutations designed to disrupt the HAP2 trimer interface as informed by the X-ray structure^19^, block appearance of SDS-resistant trimers both in vitro and in vivo, and block gamete fusion. Moreover, *minus* gametes fail to form SDS-resistant HAP2 trimers when mixed with fusion-defective *plus* gametes that lack the *plus*-specific membrane attachment protein, FUS1. Even though gametes expressing a HAP2 mutant lacking hydrophobic residues in its fusion loop are incapable of fusion with WT plus gametes, HAP2 still re-organizes into trimers, indicating that FUS1-based interactions between the membranes activate HAP2 for fusion by converting it into a form that can transit to its post-fusion form independently of target membrane insertion. Thus, the gamete membrane fusion reaction is driven by a fusogenic conformational change of HAP2 into trimers, the membrane interaction capacity of the fusion loop is required to couple trimer formation to bilayer merger, and species-specific membrane attachment catalyzes the transition.

## Results

### During the gamete membrane fusion reaction, HAP2 undergoes a fusogenic conformational change to become an SDS-resistant trimer

**Fig. 1:**
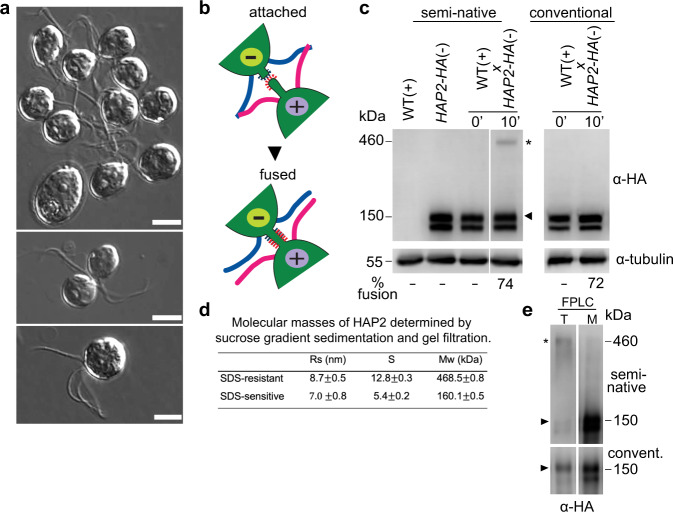
During gamete fusion, HAP2 rearranges into a trimer. **a** Differential interference (DIC) microscopic images of p*lus* and *minus* gametes that been mixed together for ~5 min followed by gentle addition with minimal agitation of an equal volume of 5% glutaraldehyde. Clusters of gametes adhering by their cilia (upper panel). A pair of gametes interacting at their apical ends during mating structure adhesion and fusion (middle panel). A quadri-ciliated zygote (lower panel). Scale bars, 5 $\mu$m. This experiment was repeated 5 times. **b** Illustration of mating structure adhesion and fusion. **c** Upon gamete fusion, an SDS-resistant HAP2 trimer appears that is stable to semi-native SDS-PAGE conditions. *HAP2-HA minus* gametes were mixed with WT *plus* gametes for 10 min and subjected to conventional SDS-PAGE (right panel) or semi-native SDS-PAGE (left panel) followed by immunoblotting with an anti-HA antibody. In the 0-min samples, the cells were suspended in sample buffer and then mixed. The percentage of gametes that fused to form quadri-ciliated cells is indicated below the blots. At least 200 cells were counted. The trimer band is indicated by an asterisk and the monomer band is indicated by an arrowhead. **d** Determination of HAP2 molecular mass by use of sucrose gradient separation and gel filtration of immunopurified fh1-HAP2-HA (see below for information about the *fh1* mutant). Results are means of 3 independent biological replicates whose peak fraction positions were used to calculate the monomer and oligomer sedimentation coefficients and Stokes radii. **e** Semi-native (upper panel) and conventional (lower panel) SDS-PAGE analyses of peak trimer (T) and monomer (M) gel filtration fractions of immunopurified fh1-HAP2-HA. The experiment was repeated 3 times with similar results.

Gamete fusion in *Chlamydomonas* is the culmination of a series of complex cellular interactions that are initiated when naive mating type *minus* and mating type *plus* gametes are mixed together. Within seconds, the gametes bind to each other by their cilia (Fig. 1a, upper panel)^33^ using the adhesion receptors SAG1 on *plus* cilia and SAD1 on *minus* cilia. Ciliary adhesion activates a signaling pathway that brings about cellular modifications (gamete activation) that prepare the gametes for fusion^34,35^. A latent, cell wall-associated metalloprotease is converted to an active form that degrades and releases the cell wall, and the gametes generate apically-localized tubular membrane protrusions–the activated mating structures–between their two cilia. The *plus* mating structure is a 50-nm diameter, ~3 μm long, actin-filled microvillus^36–38^. The *minus* mating structure is of a similar diameter, lacks actin, and is ~1 μm long^37,39^.

During receptor-mediated ciliary binding between the highly motile *plus* and *minus* gametes, the apical ends of the cells are forced against each other by the continued motility of their now tethered cilia (Fig. 1a, middle panel). The consequent, repeated collisions between the tips of the protruding mating structures between the two cilia lead to interactions between receptor proteins, (which are unrelated to SAG1 and SAD1^33^). The *plus* structure displays the single pass, transmembrane adhesion receptor FUS1^2,40^. The identity of the FUS1 binding partner on the *minus* structure has not been reported. Within 5–30 s after attachment, the tips of the two mating structures undergo HAP2-dependent fusion^12,38,41^. The newly created, membrane-enclosed tubule linking the two gametes rapidly shortens and expands as the two gametes coalesce into a single quadri-ciliated zygote (Fig. 1a, lower panel; Fig. 1b). Fertilization is rapid in *Chlamydomonas*. Zygotes are detected within 30 s after *plus* and *minus* gametes are mixed, and typically, within 5 min after mixing, over 50% of the gametes have fused^41^.

We examined the biochemical properties of HAP2 in naive *minus* gametes and newly formed zygotes. To detect HAP2, we used a strain of *minus* gametes (*hap2/HAP2-HA*) expressing a hemagglutinin (HA)-tagged form of HAP2, HAP2-HA. This is a knock-out strain, hereafter termed *HAP2-HA*, that contains both the disrupted, endogenous *HAP2* gene and a transgene encoding HAP2-HA^12^. Using conventional SDS-PAGE and immunoblotting, HAP2-HA appears as two separate bands of ~150 kDa corresponding to isomers differing in glycoslylation. Both bands migrate as HAP2 monomers, but only the form in the slower migrating band corresponds to HAP2 present at the cell surface^42^. Under these standard conditions, the ~150 kDA doublet is the only form of HAP2 detected within both naive gametes and in zygotes that form upon fusion of the *minus* gametes with wild type *plus* gametes (Fig. 1c, right panel).

In contrast, when assayed by semi-native SDS-PAGE–samples incubated with a lower concentration of reducing agent and heated at 42 °C instead of 95 °C–the HAP2-HA monomeric forms were still detected in both samples, but in the zygotes a new form of HAP2-HA appeared that migrated as a trimer (asterisk, Fig. 1c, left panel). These are conditions in which the postfusion trimers of viral class II fusion proteins, such as Semliki Forest virus E1, are SDS-resistant^43–45^. This new, distinctively different oligomeric form of HAP2 was resistant to temperatures up to 70 °C in the presence of 1% SDS and to incubation in up to 50 mM dithiothreitol (DTT) (Supplementary Fig. 1).

We also used sucrose gradient sedimentation and gel filtration^46^ to assess the state of HAP2 immuno-purified from detergent extracts of newly formed zygotes. Analysis of the fractions with semi-native SDS-PAGE and immunoblotting confirmed that HAP2 in these postfusion cells was present in two forms, monomeric and the higher molecular mass form (Supplementary Fig. 2). Calculation of the monomer molecular mass based on a sedimentation coefficient of 5.4 S and a Stokes radius of 7.0 nm Rs yielded the expected mass of ~160 kDA (Fig. 1d, and Supplementary Fig. 2). Furthermore, the analysis also demonstrated that the SDS-resistant, much more slowly migrating form of HAP2 was a trimer, its molecular mass (469 kDa) being approximately 3 times the mass of the monomer (Fig. 1d; Supplementary Fig. 2).

As expected, when the gel filtration fractions that contained the trimer band were heated to 85 °C in SDS-PAGE sample buffer containing 100 mM DTT to disrupt the trimer, only the upper form of monomeric HAP2 was detected, indicating that only the surface form of HAP2 had reorganized into the SDS-resistant trimer (Fig. 1e). Comparative mass spectrometry analysis of gel filtration samples from peak HAP2 trimer fractions and of equivalent fractions from HAP2 mutant gametes incapable of forming trimers (see below) showed that HAP2 was the only protein consistently detected in the trimer fractions (Supplementary Table 1).

### Hydrophobic residues at the trimer interface are important for appearance of the SDS-resistant trimer in vitro and in vivo, and for gamete fusion

**Fig. 2:**
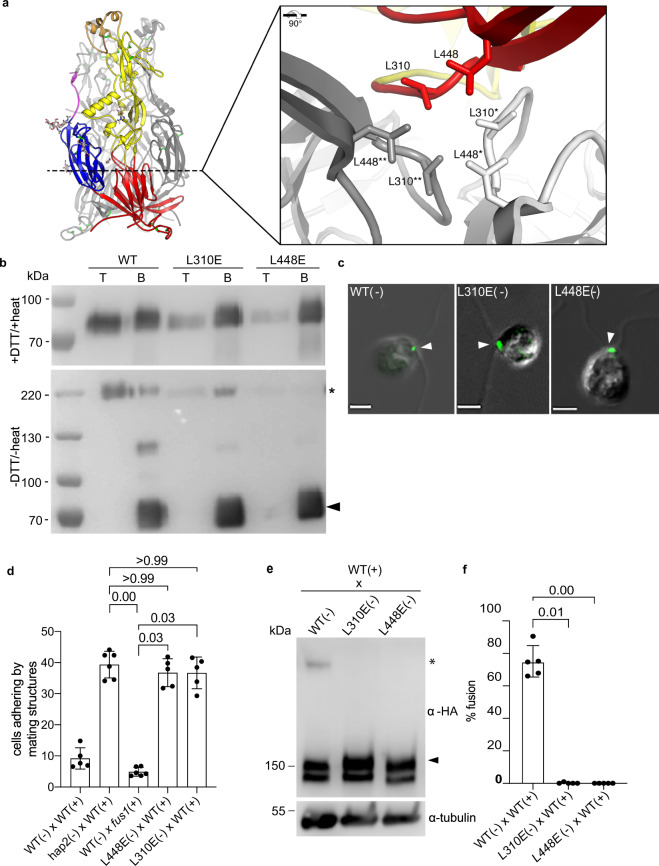
Mutations at the trimer interface prevent HAP2 trimer formation in vitro and in vivo, and block gamete fusion. **a** HAP2 trimer structure (PDB: 6E18) showing the locations of leucines 310 and 448 by the dotted line. The inset (right panel) shows a detailed view of the residues on each of the three monomers (colored red, gray, and white). **b** Immunoblots of the top (T) and bottom (B) fractions from a liposome Optiprep^TM^ co-floatation assay indicating that trimer formation was severely impaired in HAP2e-L310E and HAP2e-L448E mutants compared to WT. For the upper immunoblot, samples were prepared with 20 mM DTT and heating at 95 °C for 10 min. For the lower immunoblot, samples were prepared without heating and reducing agent. Results are representative of 5 independent experiments. **c** Indirect immunofluorescence with an anti-HA antibody showing that HAP2-HA-L310E and HAP2-HA-L448E were localized between the two cilia at the site of the *minus* mating structure, similarly to WT (arrowheads). Scale bars, 5 $\mu$m. Images are representative of at least 100 cells examined for each strain. **d** Mating structure adhesion of both *L448E* and *L310E minus* gametes with WT *plus* gametes was indistinguishable from that of the *hap2* mutant. Percentages of paired cells were determined at 10 min after mixing. *fus1 plus* gametes mixed with WT *minus* gametes were incapable of mating structure adhesion and fusion. The WT *plus* and *minus* gametes had fused by 10 min, and thus were no longer adhering. **e** Trimer formation was blocked in the *L310E* and *L448E minus* gametes. Equal numbers of *minus* and *plus* gametes were mixed for 10 min and lysates were analyzed with semi-native SDS-PAGE and immunoblotting. Lower panels in **d** and **f** are alpha tubulin loading controls. **f** Neither the *L310E* nor the *L448E HAP2* mutant was capable of gamete fusion. WT, *L310E* and *L448E minus* gametes were mixed with an equal number of *plus* gametes for 10 min followed by assessment of gamete fusion. For **d** and **f**, results are averages of at least 5 independent experiments with 200 cells counted in each sample. The Kruskal–Wallis test and Dunn’s post-test were utilized to analyze the significance of differences with *P* values labeled above the groups compared.

To assess the importance for fusion of HAP2 reorganization into trimers, we examined the effects on fusion of mutations designed to disrupt the trimer interface^19,20^. The sidechain of domain I residue L310 interacts with its counterparts from the other protomers by the threefold molecular axis at the core of the trimer, as does the sidechain of L448 (Fig. 2a). To test whether these hydrophobic interactions are important for appearance of the trimer form in vitro, we used a *Drosophila* expression system to produce soluble forms of the nearly full-length ectodomains of WT HAP2 (HAP2e), and HAP2e in which either L310 or L448 were mutated to negatively charged glutamic acid. The negative charge of this residue at the trimer core would introduce a destabilizing electrostatic repulsion effect upon trimer formation. As shown previously, WT HAP2e bound to artificial membranes as indicated by liposome co-flotation in an Optiprep^TM^ gradient (Fig. 2b, upper panel). Consistent with the previous finding that HAP2e formed trimers upon interaction with liposomes^19^, semi-native SDS-PAGE showed that the liposome-bound WT HAP2e fraction was an SDS-resistant trimer (Fig. 2b, lower panel). On the other hand, the L310E and L448E HAP2e mutants were strongly impaired in liposome co-flotation, and the band corresponding to an SDS-resistant trimer fraction was much weaker, especially for the L448E mutant (Fig. 2b). Thus, the contributions of L310 and L448 to the hydrophobic interface at the trimer core are important for HAP2e trimerization in vitro.

Complementary in vivo experiments with *hap2* *minus* gametes transformed with transgenes encoding full-length WT HAP2-HA, L310E-HAP2-HA, or L448E-HAP2-HA confirmed the in vitro results. Both the *L310E* and *L448E* mutants produced HAP2 protein at levels similar to that of the wild-type *HAP2-HA* transformant (Fig. 2c, Supplementary Fig. 3). In addition, like the WT form, the upper isoforms of both mutant forms were sensitive to trypsin, indicating that the L310E and L448E mutant HAP2 proteins were present on the gamete surface (Supplementary Fig. 3); and both were localized at the mating structure as shown by immunofluorescence analysis (Fig. 2c). Thus, neither mutation interfered with HAP2 synthesis, trafficking, or localization. On the other hand, while the *L310E* and the *L448E* *minus* gametes were fully capable of undergoing mating structure adhesion when mixed with WT *plus* gametes (Fig. 2d), the SDS-resistant trimeric form failed to appear in both, and both were incapable of fusion (Fig. 2e, f). Taken together, these results indicated that HAP2 is present in a detergent-labile form in naive *minus* gametes. During interaction with *plus* gametes, it undergoes a conformational change into an SDS resistant trimer, and the conformational change involved in this transition is essential for the membrane fusion reaction.

### FUS1-dependent attachment of the *minus* mating structure to the *plus* mating structure is required for appearance of the HAP2 trimer

**Fig. 3:**
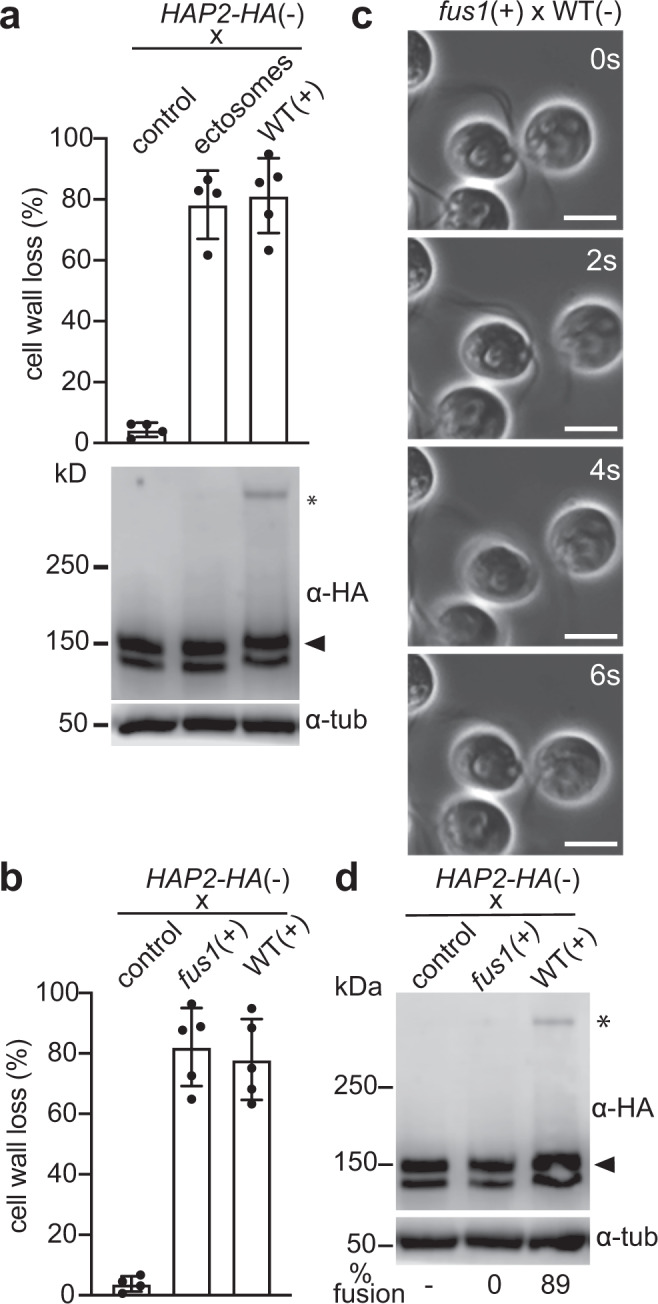
Mating structure adhesion is required for HAP2 trimer formation. **a** Ciliary ectosomes induced gamete activation, but failed to initiate HAP2 trimer formation. *HAP2-HA minus* gametes were mixed with ciliary ectosomes for 30 min and assessed for gamete activation, as indicated by a cell wall loss assay (upper panel), and for formation of SDS-resistant trimers (lower panel). The asterisk indicates the trimer band detected upon mixing HAP2-HA *minus* gametes with WT *plus* gametes for 10 min. **b** Cell wall loss in *minus* gametes mixed for 10 min with *fus1 plus* gametes or with WT *plus* gametes. Results for cell wall loss assays in **a** and **b** are averages of 5 independent experiments with samples of 3 ×10^7^ cells assessed in each. **c** Phase-contrast micrographic images from a video (Supplementary Movie 1) of an interacting *fus1 plus* gamete and a WT *minus* gamete. The mating structure-bearing apical regions of cells collided, but failed to adhere during the pushing and pulling that occur during ciliary adhesion. Scale bars, 5 μm. *n* ≥ 100. **d** *fus1 plus* gametes fail to form trimers during gamete interactions. *fus1 plus* gametes or WT *plus* gametes were mixed with *HAP2-HA minus* gametes for 10 min and subjected to semi-native SDS-PAGE and immunoblotting. The alpha-tubulin loading controls are shown on lower blots in both **a** and **d**. The blot images in **a** and **d** are representative of 3 independent experiments.

We investigated the cellular and molecular events that occur during gamete interactions that led to the appearance of the SDS-resistant trimeric form of HAP2. To determine whether activation per se of *minus* gametes in the absence of *plus* gametes was sufficient to bring about the HAP2 conformational change, we tested for appearance of SDS-resistant trimers in *minus* gametes that were experimentally induced to undergo ciliary adhesion and gamete activation without being mixed with *plus* gametes. Ciliary adhesion and the accompanying activation were induced by use of ciliary ectosomes released into the medium during prolonged adhesion of *minus* and *plus* gametes^47^. Such ciliary ectosomes contain membrane vesicles released from cilia of both types of gametes. When a sample of ectosomes is added to *minus* gametes, the SAG1-containing, *plus*-derived vesicles within the sample simultaneously bind to the cilia of several *minus* gametes, crosslinking them and leading to vigorous, ciliary-based cell-cell interactions (isoagglutination) and gamete activation^47^. By phase-contrast microscopy, these events are indistinguishable from the cell–cell interactions that occur when *plus* and *minus* gametes are mixed together. A key difference, of course, is that the *minus* gametes are incapable of undergoing homotypic mating structure adhesion or gamete fusion. As shown by an assay for cell wall loss, ectosome-induced ciliary adhesion indeed led to gamete activation (Fig. 3a, upper panel). On the other hand, immunoblotting showed that the SDS-resistant HAP2 trimeric form failed to appear (Fig. 3a, lower panel). Thus, these experiments indicated that neither ciliary adhesion-induced gamete activation itself, nor the accompanying homotypic mating structure collisions between activated *minus* gametes, which did not result in attachment, were sufficient to bring about appearance of the trimeric form of HAP2.

Given the failure of homotypic interactions to bring about the fusogenic transition of HAP2, we used mutant *plus* gametes incapable of species-specific membrane recognition to test whether heterotypic, attachment-free transient contacts between the mating structures of *plus* and *minus* gametes were sufficient to initiate formation of the trimers. We mixed HAP2-HA *minus* gametes with mutant *plus* gametes lacking the mating structure adhesion protein FUS1. In such mixtures, the gametes adhere to each other by their cilia and undergo gamete activation (Fig. 3b). Examination of live cells showed that the vigorous motility of the organelles repeatedly forced the apical ends of the *plus* and *minus* gametes with their protruding mating structures against each other, but the mating structures failed to attach^2^ (Fig. 3c). Determination of the number of gametes in pairs attached by their mating structure 10 min after mixing showed that, as expected, in the mixtures of *hap2 minus* gametes with WT *plus* gametes over 40% of the cells were in pairs, whereas in the mixtures of WT *minus* gametes with the *fus1* gametes, fewer than 4% of the cells were in pairs (Fig. 2d, above). In the mixture of WT *plus* and *minus* gametes, most of the cells had fused and HAP2 trimers had formed (Fig. 3d). In the mixture of WT *minus* gametes with *fus1 plus* gametes, however, HAP2 rearrangement into trimers was totally abrogated (Fig. 3d). These experiments indicated that FUS1-dependent mating structure adhesion was required for the fusion-driving reorganization of HAP2 into trimers.

**Fig. 4:**
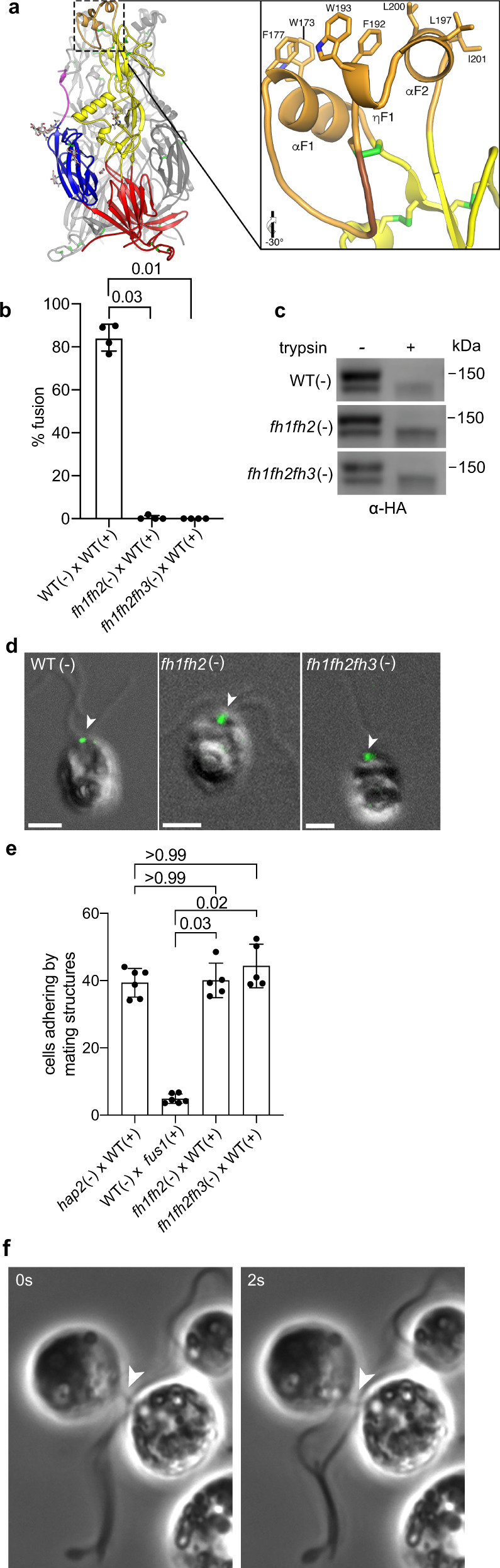
The double and triple fusion-helix mutant *minus* gametes, *fh1fh2* and *fh1fh2fh3*, express HAP2 on the cell surface at the mating structure, but fail to undergo fusion. **a** Left panel: Structure of *Chlamydomonas* HAP2 showing the overall domain organization. The fusion helices are at the top of the structure. Right panel: Higher magnification view of the fusion helices with the hydrophobic residues in each of the helices that were mutated depicted in a stick format. (ii) Amino acid sequence of the HAP2 fusion surface with the residues mutated to alanine shown in red. **b** Neither the *fh1fh2* nor the *fh1fh2fh3* HAP2 mutants were capable of gamete fusion. WT and *hap2* mutant *minus* gametes were mixed with an equal number of *plus* gametes for 10 min followed by assessment of gamete fusion. Results are averages of 4 independent experiments with 200 cells counted in each sample. **c** Protease-sensitivity assays showed that HAP2 in the *fh1fh2*, and *fh1fh2fh3 minus* gametes was located on the cell surface. Results are representative of 5 independent experiments. **d** Immunofluorescence of WT, *fh1fh2*, and *fh1fh2fh3 minus* gametes showed that the mutant HAP2s were localized similarly to WT. Scale bars are 5 $\mu$m. **e** Mating structure adhesion assays of *fh1fh2*, and *fh1fh2fh3 minus* gametes (averages of at least 5 independent experiments with 200 cells counted in each sample. These assays were performed along with those of Fig. 2D, and have in common the WT x *fus1* data). For **b** and **e**, The Kruskal-Wallis test and Dunn’s post-test were utilized to analyze the significance of differences with P values labeled above the groups compared. **f** Still images from a video (Supplementary Movie 2) of an *fh1fh2fh3* fusion helix *minus* mutant undergoing mating structure attachment with a WT *plus* gamete. The adhesive interaction between the *plus* and *minus* mating structures, which appear as a single, thin strand (arrowheads) connecting the two gametes, resists the pulling forces generated by ciliary motility.

An important consequence of mating structure adhesion is the approach of the two membranes to within ~10–15 nm of each other^12,48^, which, based on the length of crystallized post-fusion HAP2e (10 nm), is close enough for the fusion loops of HAP2 to be able to interact with the *plus* membrane. This closeness, and the results that interaction of the fusion loops of soluble HAP2e monomers with artificial membranes initiated conversion to the trimer (Fig. 2)^19,28^, raised the possibility that the fusion loop on the prefusion form of HAP2 was available for interaction with the membrane of the *plus* gamete and that it was the membrane interaction alone that initiated trimer formation. The fusion loop of HAP2 contains 3 fusion helices, αF1 (α1), ηF1 (η1), and αF2 (α2), at the tip of domain II, and each helix projects sets of hydrophobic residues positioned to interact with the membrane of the mating structure on the *plus* gamete (Fig. 4a)^20,28^. Previous in vitro studies showed that mutation of the hydrophobic residues to alanines in the first and second fusion helices (αF1 and ηF1) of HAP2e rendered it incapable in inserting into liposomes^28^. To test whether the hydrophobic residues of the HAP2 fusion loop were required for trimer formation, we generated *hap2* minus gametes expressing HAP2-HA with the same double helix mutations, *fh1fh2*, and we also generated an *fh1fh2fh3* mutant, in which the hydrophobic residues in all three of the fusion helices had been changed to alanine.

**Fig. 5:**
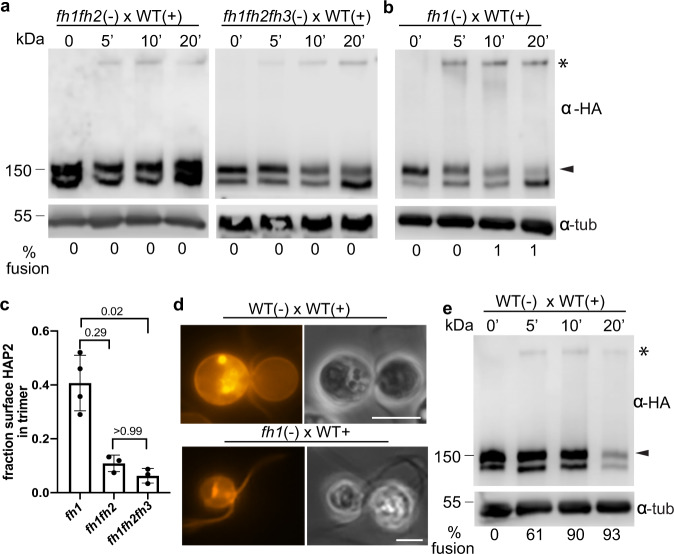
Fusion helix membrane interaction made possible by mating structure attachment promotes trimer formation and trimer formation ceases when adhesion ceases. **a**, **b** Semi-native SDS-PAGE immunoblots showing HAP2 trimer formation in fusion helix mutant *minus* gametes after mixing with *plus* gametes for the times indicated. **c** The fraction of surface HAP2 forming trimers was strongly reduced in *fh1fh2* and *fh1fh2fh3* compared to *fh1* gametes after mixing with WT *plus* gametes. Signal intensities of the trimer band and the upper monomer band at 5 min time points from at least three independent experiments were quantified by Image Studio Lite (LI-COR Biosciences). The Kruskal-Wallis test and Dunn’s post-test were utilized to analyze the statistical differences among mutants. P-values are indicated above the corresponding columns. **d** *fh1* *minus* gametes mixed with WT *plus* gametes failed to undergo hemifusion. WT *plus* gametes were incubated with membrane dye PKH26, washed into N-free medium, and mixed with the indicated *minus* gamete mutants for at least 20 min. Left panels are fluorescence images and right panels are phase-contrast images. At least 200 pairs of cells were observed with similar results. Scale bars, 5 μm. **e** Semi-native SDS-PAGE immunoblot showing HAP2 trimer formation in WT *minus* gametes after mixing with WT *plus* gametes for the indicated times. % fusion is shown under the lanes. The blot images in **a**, **b**, and **e** are representative of at least 3 independent experiments.

As expected, based on the in vitro *fh1fh2* HAP2e results and on in vivo results with single fusion helix mutants^19,20,28^, fusion by the *fh1fh2* and *fh1fh2fh3* *minus* gametes was completely blocked (Fig. 4b). Furthermore, protease-sensitivity assays with live gametes showed that, like the gametes in the previously described HAP2 single mutants^20^, the double and triple mutants expressed HAP2 at the surface of the mating structures (Fig. 4c, d), and both mutants exhibited high levels of mating structure adhesion, similar to *hap2*^20^ (Fig. 4e). Strikingly, when mixed with WT *plus* gametes, both the *fh1fh2* and *fh1fh2fh3 minus* gametes formed trimers (Fig. 5a, Supplementary Fig. 4). These results indicated that FUS1-dependent gamete adhesion signaled a change in HAP2, rendering it susceptible to undergoing a conformational change to SDS-resistant trimers in the absence of capacity for insertion of the non-polar fusion loops into the membrane of the *plus* gamete.

Despite being non-functional for fusion, a fusion loop with partial capacity for interaction with the *plus* membrane still could have promoted the conformational change of HAP2 upon mating structure adhesion. We tested this idea using *fh1* gametes, which express a HAP2 with a full complement of hydrophobic residues in 2 of its 3 fusion helices (ηF1 and αF2), but are incapable of fusion^20^. In vitro studies showed that, although the capacity for liposome insertion was reduced 5-fold compared to WT, *fh1*HAP2e was still capable of membrane interaction^28^. Notably, when the *fh1 minus* gametes were mixed with WT *plus* gametes, they underwent robust trimer formation even at 5 min, and by 20 min the surface isoform of the HAP2 doublet was noticeably diminished (Fig. 5b). Quantitative assessment of immunoblots from multiple, independent experiments indicated that 42% of fh1-HAP2-HA on the surface was in the form of trimers 5 min after mixing, significantly more than had reconfigured in the *fh1fh2fh3* triple mutant (6%, Fig. 5c). Thus, the membrane interaction sites in the fusion loop promote HAP2 trimer formation during mating structure adhesion.

Given the extensive trimer formation in these experiments with the *fh1* gametes, it was possible that the *plus* and *minus* membranes had undergone hemifusion, an intermediate step in a membrane fusion reaction in which the inner leaflets of the two interacting lipid bilayers remain separate, but the outer leaflets become continuous and undergo lipid mixing^49^. To test for hemifusion, we labeled WT *plus* gametes with the fluorescent lipid analog, PKH26, mixed them with WT and *fh1 minus* gametes, and used fluorescence microscopy to determine whether PKH26 could be detected in the *minus* gametes. When the PKH26-tagged *plus* gametes were mixed with WT *minus* gametes, fusion occurred rapidly, and lipid exchange was readily detectable in all pairs examined^12^ (Fig. 5d). On the other hand, after examining at least 100 pairs of cells in three independent experiments, we failed to detect lipid transfer in any. Thus, none had progressed to hemifusion (Fig. 5d), and, therefore, reorganization of HAP2 to trimers in the mutants was entirely non-productive. Taken together, the results above suggested that FUS1-dependent mating structure attachment initiated transition of HAP2 from its prefusion form to a form capable of undergoing the normally fusogenic conformational reorganization into trimers. The conformational reorganization progressed rapidly with HAP2 with partial capacity for lipid interaction, but trimer formation failed to merge the gamete membranes if the fusion loop lacked its full complement of hydrophobic residues.

In an independent approach to examine the function of mating structure attachment in appearance of the trimer, we mixed WT *minus* and WT *plus* gametes together and determined whether the trimer continued to appear after attachment ceased, i.e., after the tips of the mating structures had fused. The new topology created by transformation of the two protruding cellular extensions into a single, short tube enclosed by a continuous membrane would make unlikely any new interactions between FUS1 proteins and their now adjacent *minus* binding partners. Analysis of immunoblots showed that in contrast to the *fh1* gametes, in which ~40% of the surface HAP2 appeared in the trimer band at 10 min after mixing, only a small portion of HAP2, ~8%, had reorganized into the trimer form in the WT mixtures at 10 min when fusion was essentially complete (90%; Fig. 5e). That such a small portion of HAP2 was required for fusion at first might seem surprising, but this is the first time in any eukaryotic system that it has been possible to quantify the amount of a fusogen required for a cell-cell fusion event. Studies on viral fusion show that at least two adjacent sets of monomers reconfiguring trimers are required to drive fusion^24^. If the same is true here, a high concentration of HAP2 might be required on the surface of the *minus* mating structure to couple trimer formation to bilayer merger, but only a small number of trimers might be necessary for gamete fusion. Reflecting results from previous experiments, which detected only total HAP2^42^, at 20 min after mixing, substantial amounts of both the monomeric and trimeric forms of HAP2 were lost as part of a block to polygamy (Fig. 5e). Taken together, these results indicated that reorganization of HAP2 molecules on *minus* gametes into trimers was finely regulated in time and space by a FUS1-dependent mechanism, and when FUS1-mediated attachment ceased upon fusion, HAP2 conversion ceased.

## Discussion

We investigated the molecular mechanisms that underlie gamete membrane fusion by the eukaryotic class II fusion protein HAP2 during fertilization in *Chlamydomonas*. We found that gamete fusion was driven by a biochemical change in state of HAP2 during gamete interactions. In naive gametes, the protein was detected only as a monomer in our buffer conditions. Moreover, species-specific membrane attachment initiated conversion of prefusion HAP2 into trimers and the capacity for membrane interaction conferred by the hydrophobic residues of the HAP2 fusion loop promoted trimer formation and was required to couple trimer formation to bilayer merger. In WT gametes, trimer formation ceased concomitantly with the change in membrane topology that accompanied fusion of the mating structures, leaving more than 90% of prefusion HAP2 unconverted.

Multiple, independent methods documented formation of HAP2 trimers during gamete fusion. Gel filtration, velocity sedimentation, and semi-native SDS-PAGE experiments demonstrated that a new, oligomeric form of HAP2 appeared during fusion (Fig. 1b, d; Supplementary Fig. 2). The SDS-resistance of the oligomer was consistent with the properties of postfusion trimers characteristic of several viral class II fusion proteins, and with trimers formed in vitro by experimentally produced HAP2e (Fig. 2b). Mass spectrometry analyses showed that the trimer-containing gel filtration fractions of immuno-purified HAP2 trimers were composed of HAP2 (Supplementary Table 1). And, the molecular mass of the SDS-resistant trimer determined by gel filtration and velocity sedimentation was three times that of non-rearranged HAP2, which behaves as a monomer in our buffer conditions (Fig. 1d). Furthermore, complementary structure-guided mutational analyses in vivo and in vitro demonstrated that trimer formation was not simply an epiphenomenon associated with fusion, but was essential for fusion (Fig. 2). Separately mutating either of two residues in the central core of the HAP2e structure impaired trimer formation in vitro, and although the corresponding mutations in vivo were without any observable effect on HAP2 expression or localization, they completely blocked trimer formation and they completely blocked gamete fusion.

In spite of the functional and structural similarities between HAP2 and the viral class II proteins, our findings and consideration of the circumstances of gamete fusion suggest that the mechanisms for regulating the irreversible transition from the metastable prefusion form into the highly stable postfusion form have important differences from those of viral class II fusion proteins. In class II viral systems, virus interaction with the plasma membrane of target cells fails to initiate trimer formation. Only after the virions have undergone endocytosis and arrive in the acidic milieu of the endosome is the fusogenic conformational transition initiated^25^. In *Chlamydomonas*, initiation of the transition occurs at the cell surface in the neutral pH milieu of the culture medium. The adhesion receptor-based mechanism shown here for initiation of trimer formation ensures that the frequent and multiple collisions of the *minus* mating structure with the plasma membranes of the cell bodies of *plus* and *minus* gametes leads to fusion only at membrane sites specialized for fusion and bearing FUS1. A reasonable prediction from our results is that species-specific attachment proteins in gametes of other HAP2-expressing organisms, including the human malaria pathogens *Plasmodium falciparum* and *Plasmodium vivax*, also will be essential for regulating conversion of the prefusion forms of their HAP2s into fusion-driving trimers.

**Fig. 6:**
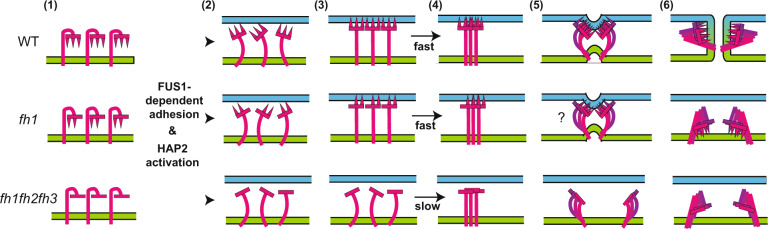
Working model for transition of prefusion HAP2 into trimers in WT and fusion helix mutants. (1) Prefusion HAP2 with the 3 fusion helices of the fusion loop in a cryptic form on the membrane of the *minus* mating structure. The blue lines represent the membrane on the *plus* mating structure and the green lines represent the membrane on the *minus* mating structure. The magenta line with a trident on top represents one HAP2 monomer. Each spike of the trident represents the hydrophobic residues of one of the fusion helices. *fh1* lacks hydrophobic residues in one fusion helix (middle panel) and the hydrophobic residues are missing in all 3 of the fusion helices of *fh1fh2fh3* (lower panel). (2) FUS1-dependent interaction with the membrane of the *plus* mating structure (blue bar) allows HAP2 to transition to an extended monomeric intermediate with an exposed fusion loop. (3) The fusion loop of monomeric HAP2 interacts with the membrane of the *plus* mating structure, which promotes trimer formation in WT and *fh1* but not in *fh1fh2fh3*. (4) The trimeric, extended intermediate of HAP2 bridges the two membranes. (5) The extended trimer folds back, dragging the two membranes close. (6) Formation of the highly stable postfusion trimer is coupled with membrane merger in WT but not in *fh1* or *fh1fh2fh3*.

The requirement for FUS1-dependent attachment between *plus* and *minus* mating structures to initiate HAP2 trimer formation explains the previously reported inability of WT *minus* gametes to fuse with *plus* gametes lacking FUS1^2,40^, but also raises the question of the molecular function of attachment in initiation of trimer formation. Our findings that a HAP2 whose fusion loop had partial capacity for membrane interaction formed trimers at a faster rate than that of a HAP2 devoid of hydrophobic residues in its fusion loop (Fig. 5) suggest that one function of attachment is to bring the membranes of the two gametes close enough for HAP2 to reach across the gap and interact with the membrane of the *plus* mating structure. On the other hand, the triple fusion helix mutant form of HAP2 (*fh1fh2fh3)*, which was completely blocked in liposome insertion in vitro, was capable of undergoing some (non-fusogenic) rearrangement into trimers during mating structure attachment in vivo (Fig. 5a). A simple model is that adhesion receptor-mediated attachment initiates a change in prefusion HAP2 that allows trimer formation, and interaction of the fusion loop with the *plus* membrane enhances trimer formation and is essential to couple trimer formation to bilayer merger (Fig. 6).

In the viruses that use class II fusion proteins and undergo fusion only in the endosome, the hydrophobic fusion loops of the proteins are shielded within prefusion homo- or heterodimers in the viral envelope and are not accessible until the protein complexes are disrupted by the low pH of the endosome. If the fusion helices in prefusion HAP2 are similarly shielded in labile and as yet uncharacterized protein complexes, attachment could directly or indirectly initiate changes in HAP2 that are permissive for trimer formation and that unshield the fusion loop. Such a mechanism would add a new twist to the challenges of understanding the evolution of eukaryotic fusion proteins^8,22^, and would be similar to that of HIV and some coronaviruses that use class I fusion proteins. These viruses fuse at the cell surface, and the fusogenic conformational changes of their class I proteins are initiated by interactions between viral receptor binding proteins and membrane receptors on the surface of the target cell^24,50^.

We should note that our evidence for the unilateral mechanism of HAP2-driven membrane fusion in *Chlamydomonas* is not consistent with earlier bilateral models proposed for HAP2 action that were based on studies with *Arabidopsis thaliana* HAP2 expressed in vertebrate cells^23,30^. In those models, formation of HAP2 homotrimers composed of HAP2 monomers from each cell or of heterotrimers composed of HAP2 from one cell and an unidentified protein from the other cell was proposed, but not examined biochemically. The high levels of HAP2 expressed in the vertebrate cells used in those experiments, along with the presence of the hydrophobic HAP2 fusion loops in an unknown state of shielding at the plasma membrane, even makes it possible that the fusion observed was not mediated by trimer formation. The ability to identify and quantify trimer formation described here should be useful to examine HAP2 mechanisms in other organisms, to examine the mechanism that maintains HAP2 in its metastable form before fusion, and to investigate the coupling of species-specific attachment to initiation of the fusogenic rearrangement of HAP2.

In summary, the ease of culturing quantities of wild type and mutant strains of *Chlamydomonas* gametes sufficient for biochemical studies on HAP2, along with the ability to experimentally separate membrane adhesion and lipid bilayer merger from the many other events that comprise the complex, multistage process of fertilization have made possible mechanistic studies of the gamete membrane fusion reaction. The work reported here provides evidence that the biochemical reaction at the core of fertilization in eukaryotes across kingdoms is a fusogenic conformational rearrangement of eukaryotic class II fusion protein HAP2, whose substrate is two lipid bilayers and whose product in a single lipid bilayer. Before fusion, the protein is in an as yet uncharacterized conformational state that is detected as a monomer in the detergent-containing buffers used here. Upon the rearrangements that occur during fusion, HAP2 becomes the SDS-resistant trimer. The finding that species-specific adhesion initiates the fusogenic reorganization of HAP2 also provides a new dimension to our understanding of the function of species-specific gamete adhesion during fertilization in HAP2-bearing organisms. Not only does adhesion allow sex cells to recognize each other, but it is also the gateway to activation of the fusion machinery.

## Methods

### Cells and cell culture

Wild-type *Chlamydomonas* strains *21gr* (mating type *plus*; CC-1690), *40D4* (*hap2* mating type *minus* mutant)^48^, and *fus1*(*fus1* mating type *plus* mutant; CC-2062), are available from the *Chlamydomonas* culture collection. Generation of *HAP2-HA* (*HAP2-HA* plasmid-rescued *hap2-2* mating type *minus*) and *HAP2-HA* fusion loop mutants *fh1*, *fh2* and *fh3* (mating type *minus*) was described previously^20^. Cells were grown vegetatively in TAP medium under a 13:11 light-dark cycle at 25 °C as described previously. Gametogenesis was induced by transferring vegetative cells to N-free medium followed by overnight agitation on a shaker in continuous light. Assays for mating structure adhesion and for gamete fusion were carried out as described previously^20^.

### Plasmid construction and transformation into *Chlamydomonas*

Modified versions of the Psil-NcoI restriction fragment of *HAP2-HA* plasmid *pJJ1*^20^ containing a paramomycin resistance gene, *aphVIII* were synthesized (Genscript, https://www.genscript.com/) and used to replace the original fragment to generate the following plasmids: *pJJ5*, which encodes HAP2-HA-W173A/F177A/F192A/W193A (*fh1fh2* for short); *pJJ6*, which encodes HAP2-HA-W173A/F177A/F192A/W193A/L197A/L200A/I201A (*fh1fh2hf3* for short); *pJJ7*, which encodes HAP2-HA-L310E; *pJJ8*, which encodes HAP2-HA-L448E. Codon GCC was used for alanine and GAG was used for glutamic acid. Plasmid sequences were confirmed by DNA sequencing and the plasmids were introduced into the *40D4 hap2* mutant using electroporation^51^. Colonies that grew on paromomycin plates were screened by PCR for the presence of the transgenes with primers P18 (5′-CCGATAATGCCTGAACACAATTCCA-3′ and P19 (5′- GTATGTCCAGTGGGTCGCTCCAGAAG-3′). HAP2-HA expression was detected by immunoblotting with a rat anti-HA antibody (3F10, Roche).

### Mating structure adhesion assay

The ability of gametes to adhere by their mating structures was determined by phase-contrast microscopy^20^. 10 min after equal numbers of *plus* and *minus* gametes (2 ×10^7^ cells/ml in N-free medium) were mixed together, aliquots were fixed with an equal volume of 5% glutaraldehyde, ciliary adhesions were disrupted by gently pipetting 10 times with a 1 ml pipette tip, and the percent of cells present as pairs was determined by microscopy.

### Determination of cell fusion

The ability of gametes to fuse to form zygotes, which have four cilia as opposed to unfused gametes, which have two cilia, was quantified by phase-contrast microscopy^20^. *Plus* and *minus* gametes in equal numbers were mixed for 10 min, fixed with an equal volume of 5% glutaraldehyde, and the percent of single cells that had fused to form quadri-ciliated zygotes was determined (2 × number of quadri-ciliated cells/([2 × number of quadri-ciliated cells + number of single gametes] × 100)^12^. At least 200 cells were counted for each sample. To prepare images of interacting gametes, equal numbers of *plus* and *minus* gametes were mixed together for 5 *minus*, fixed by addition of an equal volume of 5% glutaradehyde, and briefly agitated. The cells were examined by DIC microscopy with a Zeiss Axiovert 135 inverted microscope using a Plan-Neofluar Pol 63X objective (Numerical aperture, 1.25). The microscope is coupled with a PIKE F032B monochrome camera (Allied Vision Technology) controlled by StreamPix software for imaging.

### Indirect immunofluorescence

Indirect immunofluorescence was performed as previously described^20^. Briefly, after fixing gametes in ice-cold methanol for 20 min, samples were blocked with goat serum and probed with 1/100 rat anti-HA (Sigma). Samples were then washed with 1x PBS, stained with 1:400 Alexa Fluor 488-conjugated goat anti-rat IgG (Invitrogen), and mounted using Fluoromount-G prior to visualization on a Leica SP5 X Laser Scanning confocal microscope with a Leica 63x/1.4 NA oil objective lens controlled by Leica LASAF software.

### Lipid staining with PKH26

Lipid staining with PKH26 was performed as previously described^12^ with minor modifications. Briefly, pre-activated WT *plus* gametes (2 ×10^8^ cell/ml in N-free medium) were mixed with an equal volume of staining solution containing PKH26 (4 ${\rm{\mu }}{\rm{M}}$ in Diluent C, PKH26 Red Fluorescent Cell Linker Kits for General Cell Membrane Labeling, Sigma-Aldrich) for 10 min at room temperature. Cells were washed three times with N-free medium by centrifugation. The labeled gametes were mixed with WT or mutant *minus* gametes and examined by epi-fluorescence and DIC microscopy with a Zeiss AxioObserver Fluorescence Microscope fitted with the Plan-Neofluar 100x/1.3 NA oil objective lens. Images were obtained and processed by Zeiss Zen blue imaging software.

### SDS-PAGE and immunoblotting

Gametes (1 ×10^7^ cells in N-free medium) were harvested and resuspended in 100 ${\rm{\mu }}$l HMDEK buffer (10 mM HEPES, pH 7.2, 5 mM MgSO4, 1 mM EDTA, and 25 mM KCl, Protease inhibitor cocktail [Sigma], 1 mM Na_2_VO_3_, 5 mM NaF, 0.2 mM PMSF). For analysis by semi-native SDS-PAGE, the samples were mixed with semi-native SDS-PAGE sample buffer (100 mM Tris, pH 6.8, 8% glycerol, 0.1 mg/ml Bromophenol, 2% SDS, 10 mM DTT) and incubated at 42 °C for 5 min. For conventional SDS-PAGE, the semi-native SDS-PAGE buffer was used, but with a final concentration of DTT of 100 mM, and samples were heated to 95 °C. Protein transfer and immunoblotting were as described previously^2^ with the following modifications. Polyacrylamide (7%) tris-glycine gels were used with the exception of the blot in Fig. 1b, which used a NuPAGE™ 7% Tris-Acetate ProteinGel (Invitrogen). Rat anti-HA from Roche (3F10, catalog No. 11867423 001) was used as the primary antibody and HRP-conjugated goat anti-rat IgG antibody (catalog No. AP136P, Sigma). was used as the secondary antibody. Blot membranes were scanned with a C-digit blot scanner (Li-Cor Biosciences) and the images were processed by Image Studio software (Li-Cor Biosciences) and Photoshop.

### Immunoprecipitation

Immunoprecipitation of HAP2-HA was performed as described previously^42^ with minor modifications. Briefly, *fh1 minus* gametes (2 ×10^9^ cells in N-free medium) were mixed with an equal number of WT *plus* gametes (final volume 40 ml) for 20 min. The *fh1* cells were used because a large portion of their surface HAP2 transitions to SDS-resistant trimers (Fig. 5). The cells were harvested by centrifugation at 3000 g for 5 min and disrupted by incubation in 5 ml RIPA buffer (25 mM Tris-HCl pH 7.6, 150 mM NaCl, 1% NP-40, 0.5% sodium deoxycholate, 0.1% SDS) containing protease inhibitor cocktail (Sigma-Aldrich), 1 mM Na_2_VO_3_, 5 mM NaF, 0.2 mM PMSF) with gentle rotation for 30 min at 4 °C. The cell lysate was then centrifuged at 12,000 × *g*, 4 °C, for 30 min and the supernatant was incubated with monoclonal rat anti-HA affinity matrix (3F10, Roche, catalog No. 11867423001) with gentle rotation for 4 h at 4 °C. The matrix was collected by centrifugation at 12,000 × *g* for 30 s and washed two times with RIPA buffer and three times with FPLC buffer (20 mM tris-HCl (pH7.5), 150 mM NaCl, 5% Glycerol, 0.1 % triton X-100) containing protease inhibitor cocktail 4 °C. 2 volumes of 1 mg/ml HA peptide in FPLC buffer with protease inhibitor cocktail were incubated with the matrix at 37$\,^\circ {\rm{C}}\,$for 10 min to elute HAP2-HA.

### Gel filtration and sucrose gradient centrifugation

The immuno-purified HAP2-HA was subjected to analysis by both gel filtration and sucrose gradient centrifugation. For gel filtration, a 50 µl sample was loaded onto a Superdex 200 10/300 GL column (GE healthcare) equilibrated with FPLC buffer. Separations were performed at 20 °C with a flow rate of 0.4 ml/min using a BioLogic DuoFlow 10 FPLC System (Bio-Rad)^20^. Fractions (0.5 ml) were collected beginning 10 min after the injection and 25 µl portions were subjected to semi-native SDS-PAGE and immunoblotting as described above. For sucrose gradient centrifugation, 4 ml, 8–30% continuous sucrose gradients in FPLC running buffer (20 mM Tris-HCl pH 7.5, 150 mM NaCl, 0.1% triton X-100, 5% glycerol, protease inhibitor cocktail) were made using a manual gradient maker. A 50 µl sample was loaded onto the gradient followed by centrifugation at 122,000 x *g* for 17 h using a MLS-50 swinging-bucket rotor (Beckman Coulter). 21 fractions (200 µl) were taken from each gradient^52^. HAP2-HA was detected by semi-native SDS-PAGE and immunoblotting.

### Liquid chromatography-tandem mass spectrometry

Peak gel filtration fractions that contained purified trimeric HAP2 were subjected to acetone precipitation^53^. Proteins were re-dissolved in 25 µl 5% SDS, reduced with DTT, alkylated with iodoacetamide (IAA), acidified with 3 µl of 12% H_3_PO_4_, and 165 µl of binding buffer (90% MeOH, 10% 1 M triethylamine ammonium bicarbonate) was added to the protein samples to form micelles. The colloidal solution was loaded into an S-Trap (Protifi, Huntington, NY) micro spin column, and washed three times with 150 µl binding buffer. Trypsin/LysC mix (1 µg, Promega) was added to the S-Trap and incubated at 45 °C for 3 h. Tryptic peptides were eluted, dried under vacuum, and dissolved in 20 µl 0.1% formic acid. Standard peptide solution (50x diluted iRT, 1 µl) was added.

The entire tryptic digest (21 µl) was injected for LC-MSMS analysis. Peptides were loaded into a trap column (Agilent Zorbax 300SB-C18, 0.3 × 5 mm) at 100% solvent A (0.1% formic acid) with a flow rate of 5 µl/min. Peptides were then separated using a Thermo Scientific Accalaim PepMap™ 100, 2 µm, 100 Å, 75 µm × 250 mm Nano column. A linear gradient of 1–40% solvent B (75% ACN 0.1% formic acid) was run for 120 min with a flow rate of 300 nl/ min. The eluents were analyzed with an Orbitrap Fusion Lumos mass spectrometer (Thermo Scientific). Precursor masses were detected in the Orbitrap at R = 120,000 (m/z 200). Fragment masses were detected in a linear ion trap at unit mass resolution. Data-dependent MSMS was carried with a top speed setting and cycle time was 3 s. Dynamic exclusion time was 30 s.

The acquired data were processed with Proteome Discover (Thermo scientific, version 2.2) and searched against the *Chlamydomonas reinhardtii* proteome database from Phytozome (v5.6, https://phytozome.jgi.doe.gov/pz/portal.html#!info?alias=Org_Creinhardtii). Cysteine carbomidomethylation was set as a fixed modification. Protein N-terminal acetylation, methionine oxidation, and NQ deamidation were set as the variable modifications. Precursor mass tolerance was set to be 20 ppm and later filtered to 5 ppm in report. Fragment mass tolerance was 0.6 Da. These analyses were carried out both with trimer peak fractions of *fh1-*HAP2-HA from gel filtration and with equivalent, but trimer-lacking fractions of HAP2-L310E-HA, a form that is incapable of forming trimers (Fig. 2).

### Recombinant protein expression and purification

*Chlamydomonas reinhardtii* HAP2 ectodomain (HAP2e, residues 23-592) with a C-terminal double strep-tag was purified from *Drosophila* S2 stable cell lines^19^. These cells were maintained in flasks at 28 °C in HyClone medium (GE Healthcare Life Sciences) supplemented with 50 units/mL of penicillin, 50 units/mL of streptomycin and 7 μg/mL of puromycin. Large-scale cultures for protein production were grown in spinner flasks at 100 rpm until reaching a cell density of approximately 7×10^6^ cells/ml. At this point the expression of the protein, which is under the control of the metallothionein promoter, was induced by adding 4 μM CdCl2 to the cell culture medium. At 5 days post-induction, the cells were centrifuged and the soluble ectodomain was purified by affinity chromatography from the supernatant on a StrepTactin Superflow column. The protein was further purified by SEC on a Superdex200 column equilibrated with PBS and fractions corresponding to the monomeric peak^19^ were pooled and concentrated. Mutant ectodomains at positions 310 (L310E) and 488 (L488E) were generated by standard PCR methods and they were expressed and purified as indicated for the wild-type protein.

### Liposome co-flotation experiments

Liposomes composed of DOPE (1,2-dioleoyl-sn-glycero-3-phosphoethanolamine), DOPC (1,2-dioleoyl- sn-glycero-3-phosphocholine), cholesterol and sphingomyelin in a molar ratio1/1/3/1, were prepared by the freeze-thaw and extrusion method. 1 μM protein was mixed with 1 mM freshly prepared liposomes and incubated for 1 h at 25 °C in 100 μL PBS. Samples were adjusted to a final concentration of 40% Optiprep™ (in PBS), loaded in centrifuge tubes and overlaid with 4.5 mL 20% Optiprep™ and 0.3 mL PBS. Centrifugations were performed overnight at 4 °C and 192,000 × *g* on SW55ti rotor. Top and bottom fractions of the gradient were analyzed by immunoblotting using anti-Strep tag antibody.

### Cell wall loss assay

The ability of gametes to lose their cell walls upon mixing, which is a measure of ciliary adhesion-induced signaling and gamete activation, was determined as described^54^. Briefly, *plus* and *minus* gametes (5 ×10^7^ cells/ml in N-free medium) were mixed for 10 min, aliquots were added to 1.6 volumes of ice-cold N-free medium containing 0.075% Triton-X 100 and 5 mM EDTA, briefly vortexed, subjected to centrifugation (8700 × *g* for 30 s), and the OD435 of the supernatant was determined immediately using a Nanodrop 2000 spectrophotometer (Thermo Scientific). The OD435 of a sample of similarly treated gametes that had first been disrupted by sonication (three times for 5 s each on ice (Microson XL sonicator) was used as a measure of 100% cell wall loss.

### Mating structure adhesion

After mixing equal numbers of *plus* and *minus* gametes (2×10^7^ cells/ml in N-free medium) for 10 min, cells were fixed by adding an equal volume of 5% glutaraldehyde. Ciliary adhesions were disrupted by gently pipetting cells 10 times with a 1 ml pipette tip. The numbers of cells in pairs per 200 cells for each sample were determined using phase-contrast microscopy (Zeiss).

### Gamete fusion

*Plus* and *minus* gametes in equal numbers were mixed for 5 min, fixed with an equal volume of 5% glutaraldehyde, and the percent of single cells that had fused to form quadri-ciliated zygotes was determined (2 × number of quadri-ciliated cells/([2 × number of quadri-ciliated cells + number of single gametes] × 100). At least 200 cells were counted for each sample.

### Quantification and statistical analysis

Data are presented as mean ± SD and were analyzed by Prism 8.0 (GraphPad). The Kruskal-Wallis test and Dunn’s post-test were utilized to analyze the significance of differences among different groups.

## Supplementary information

Supplementary Information

Descriptions of Additional Supplementary Files

Supplementary Movie 1

Supplementary Movie 2

## Data Availability

The published article includes all the data generated during this study. Source data are provided with this paper.

## Source data

Source Data
