# Supplementary material for: Species-specific gamete recognition initiates fusion-driving trimer formation by conserved fusogen HAP2
Source: Nat Commun. 2021 Jul 19;12:4380. doi: 10.1038/s41467-021-24613-8 (PMC8289870; doi:10.1038/s41467-021-24613-8)
Supplement: Supplementary file 1 — Supplementary Information [file 41467_2021_24613_MOESM1_ESM.pdf]

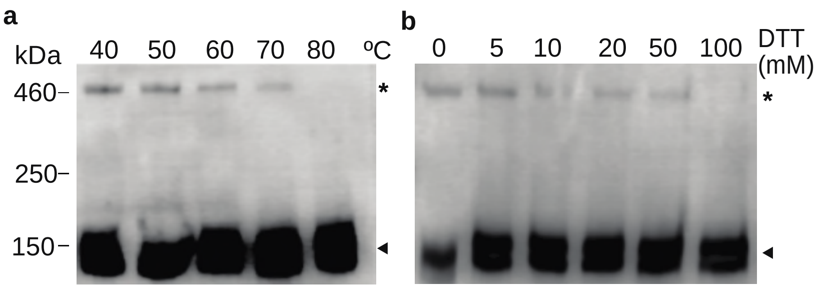

Supplementary Fig. 1. The HAP2 trimer is sensitive to elevated temperature and high concentrations of reducing agent. a *HAP2-HA minus* gametes were mixed with WT *plus* gametes for 10 *minus*, harvested, and suspended in semi-native SDS-PAGE sample buffer followed by incubation of the cell lysates at the indicated temperatures for 5 min. **b** *HAP2-HA minus* gametes were mixed with WT *plus* gametes for 10 min and incubated in semi-native SDS-PAGE sample buffer with the indicated concentrations of DTT at 40°C for 5 min. For **a** and **b**, images are representatives of three independent experiments.

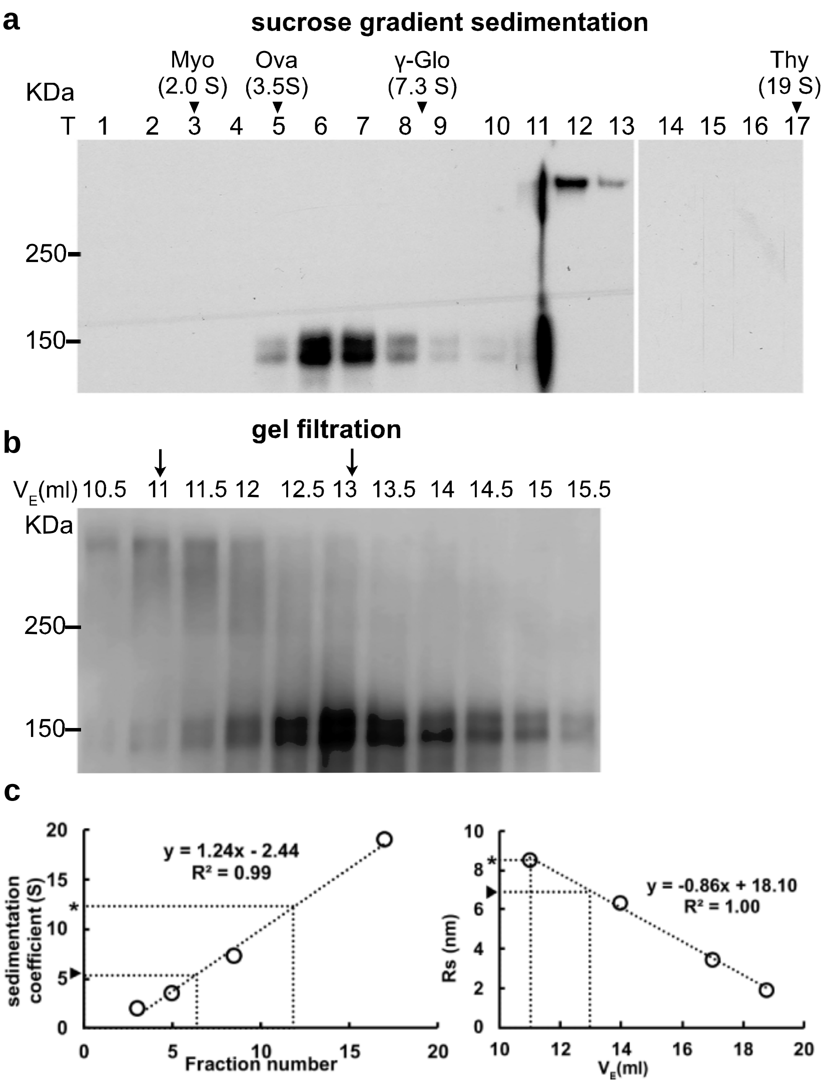

Supplementary Fig. 2. Analysis of the HAP2 SDS-resistant trimer by sucrose gradient sedimentation and gel filtration. a Immuno-purified HAP2-HA obtained from RIPA lysates of *fh1 minus* gametes mixed with WT *plus* gametes for 20 minutes was subjected to centrifugation on an 8-30% sucrose gradient and fractions analyzed by semi-native SDS-PAGE immunoblotting. The peak fractions for the protein size standards used are indicated above the blot with letters representing their identities as Myo, myoglobin; Ova, ovalbumin; γ-Glo, gamma-globulin; and Thy, Thyroglobulin. The peak sucrose gradient fraction positions for the HAP2 monomer and oligomer are indicated by black arrows. **b** Affinity-purified HAP2-HA obtained from detergent lysates of *fh1-HAP2-HA minus* gametes that had been mixed with WT *plus* gametes for 20

minutes were separated by gel filtration and analyzed by semi-native SDS-PAGE immunoblot analysis. Peak gel filtration fractions are indicated for the HAP2 monomer and the oligomer by black arrows. **c** Analysis of HAP2 molecular mass by use of sucrose gradient separation and gel filtration of immunopurified *fh1*-HAP2-HA. The linear equations and R^2 shown were used for the final estimation of the sedimentation coefficients (S) (left) and Stokes radii of standards (nm) (right) to calculate the molecular masses of HAP2 monomeric and oligomeric forms. Peak fractions of monomer (arrowheads) and trimer (asterisks) are indicated along with the corresponding S values and Stokes radii. Results are representative of one of three independent biological replicates whose peak fraction positions were averaged and used to calculate the monomer and oligomer sedimentation coefficients and Stokes radii.

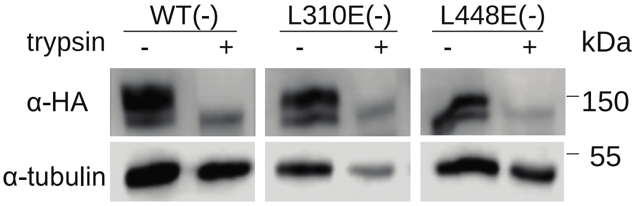

Supplementary Fig. 3. HAP2-L310E and L448E are present on the cell surface. HA immunoblotting after trypsin treatment of live cells shows that the upper forms of both the L310E and L448E HAP2 mutants were protease-sensitive, indicating that both forms were present on the cell surface. Results are representatives of five independent experiments.

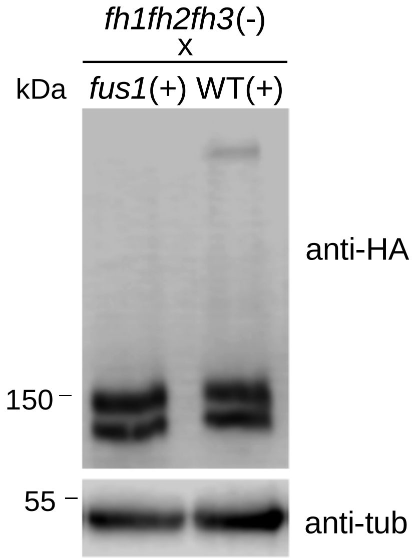

Supplementary Fig. 4. Mating structure adhesion is required for trimer formation by *fh1fh2fh3 minus* gametes. *fh1fh2fh3-HAP2-HA minus* gametes were mixed with *fus1 plus* gametes or with WT *plus* gametes for 10 minutes and analyzed by semi-native SDS-PAGE and immunoblotting. This experiment was repeated three times with similar result.

Supplementary Table 1. Peptides present in HAP2 trimer peak fraction from 2 biological replicates and in an equivalent fraction from 1 negative control (L310E).

	Peptides Run #1 (*fh1*- x *wt*+)	Peptides Run #2 (*fh1*- x *wt*+)	Peptides (L310E- x *wt*+)
**HAP2HA**	**790-816 (1); 159-185 (4)**	**874-899 (4); 286-302 (2); 914-922 (2);471-478 (2)**	**not found**
Cre11.g467650.t1.1	not found	278-297 (8)	not found
Cre12.g560150	not found	28-46 (2)	not found
Cre09.g405900.t1.1	not found	1212-1222 (2)	not found
Cre06.g263250.t1.1	not found	258-275 (4); 178-195(2); 156-177 (2)	not found
Cre06.g264600.t1.2	not found	47-56 (4)	not found
Cre02.g080600.t1.2	not found	225-240 (12); 79-89 (2)	not found
Cre13.g577050.t1.1	605-629 (1)	not found	not found
Cre03.g190450.t1.1	232-286 (1)	not found	not found
Cre08.g363874.t1.1	485-496 (1)	not found	not found
Cre17.g730483.t1.1	1026-1049 (1)	not found	not found
Cre11.g467759.t1.1	1-8 (1)	not found	not found
Cre09.g388652.t1.1	388-405 (1)	not found	not found
Cre06.g265000.t1.2	116-128 (2)	not found	not found
Cre02.g104800.t1.1	74-80 (2)	not found	not found
Cre03.g189150.t1.1	3138-3186 (1)	not found	4158-4168 (1)
Cre16.g663350.t1.2	not found	346-358 (4)	346-358 (2)
Cre13.g580900.t1.1	not found	266-274 (7)	266-274 (2); 266-272 (1)
Cre11.g467644.t1.1	not found	217-226 (3); 255-262 (1)	217-226 (4); 204-216 (1)
Cre06.g259150.t1.2	not found	124-138 (1)	124-138 (2)

*Numbers indicate the start point and the end point of the peptide. Numbers in parentheses indicate the number of times the peptide was detected.
