# Supplementary material for: Species-specific gamete recognition initiates fusion-driving trimer formation by conserved fusogen HAP2
Source: Nat Commun. 2021 Jul 19;12:4380. doi: 10.1038/s41467-021-24613-8 (PMC8289870; doi:10.1038/s41467-021-24613-8)
Supplement: Supplementary file 2 — Descriptions of Additional Supplementary Files [file 41467_2021_24613_MOESM2_ESM.pdf]

Descriptions of Additional Supplementary Files

Supplementary Movie 1. Phase-contrast microscopy video using a 100 x objective showing pairs of *fus1 plus* gametes and WT *minus* gametes whose apical ends interacted but failed to attach. This video is related to Fig. 3C.

Supplementary Movie 2. Phase-contrast microscopy video using a 100 x objective showing an *fh1fh2fh3* fusion helix mutant and a WT *plus* gamete attached to each other by their mating structures. This video is related to Fig. 4D-ii.
